# Albuminuria as a Key Factor Associated with Ambulatory Arterial Stiffness: A Hierarchical Multivariable Analysis

**DOI:** 10.3390/jcm15041498

**Published:** 2026-02-14

**Authors:** Kemal Ozan Lule, Ozge Ozsoy, Omer Yildirim, Hamit Yildiz

**Affiliations:** Internal Medicine Department, Faculty of Medicine, Gaziantep University, 27310 Gaziantep, Türkiye; ozsoyozgee@gmail.com (O.O.); yldrmomer40@gmail.com (O.Y.); drhyildiz@hotmail.com (H.Y.)

**Keywords:** AASI, ABPM, albuminuria, arterial stiffness, hypertension

## Abstract

**Background:** The ambulatory arterial stiffness index (AASI) is a non-invasive surrogate marker of arterial stiffness; however, the relative contributions of hemodynamic, cardiometabolic, and renal factors to the AASI remain incompletely understood. This study aimed to identify the independent clinical factors associated with the AASI. **Methods:** This retrospective cross-sectional study included 290 individuals aged 18–65 years who underwent ABPM between 2020 and 2024. Participants were classified as hypertensive or normotensive based on ABPM criteria. Hemodynamic parameters, cardiometabolic indices, and renal biomarkers, including the urine albumin-to-creatinine ratio (uACR), were assessed. **Results:** Associations between the AASI and clinical variables were evaluated using the following correlation analyses and hierarchical multivariable linear regression models: Model 1-1b (hemodynamic), Model 2 (hemodynamic plus cardiometabolic) and Model 3 (hemodynamic plus cardiometabolic plus renal). The AASI was significantly higher in hypertensive individuals compared with normotensive controls. In correlation analyses, the AASI was positively associated with age, systolic blood pressure parameters, atherogenic lipid indices, and uACR and negatively associated with diastolic blood pressure parameters and the estimated glomerular filtration rate (eGFR). In multivariable regression analyses, age, maximum systolic blood pressure, and maximum diastolic blood pressure remained independently associated with the AASI across models. uACR was also independently associated with the AASI in the fully adjusted model. **Conclusions:** The AASI is primarily associated with hemodynamic load and age-related vascular changes. Among non-hemodynamic factors, albuminuria demonstrated the strongest association with the AASI after multivariable adjustment. These findings suggest the potential clinical value of the AASI as a practical marker for early cardiorenal risk assessment using routine ABPM data.

## 1. Introduction

Hypertension is a major and modifiable global public health problem and is responsible for millions of deaths each year due to its cardiovascular and renal complications [1]. Prolonged arterial hypertension can lead to both structural and functional damage in target organs, particularly the cardiovascular and renal systems [2].

Although systolic and diastolic blood pressure values have traditionally been used as the primary parameters for the assessment of hypertension, these measurements do not adequately reflect arterial wall stiffness or the pressure load imposed on target organs. In contrast, the ambulatory arterial stiffness index (AASI) is a non-invasive and robust parameter derived from 24-h ambulatory blood pressure monitoring data that enables an indirect assessment of arterial stiffness [3,4]. Several studies have demonstrated that the AASI is a more sensitive indicator of arterial wall compliance than conventional blood pressure measurements. Moreover, the reported associations between the AASI and cardiovascular events, target organ damage, and all-cause mortality suggest that this index may serve as a prognostic tool in clinical practice [5,6]. In the literature, the lack of comprehensive studies evaluating hemodynamic, cardiometabolic, and renal parameters within the same multivariable models limits a clear understanding of the true clinical determinants and potential pathophysiological role of the AASI. In this context, assessing the relationship between the AASI and cardiometabolic and renal biomarkers may contribute to the early identification of hypertension-related target organ damage and improve our understanding of the potential role of the AASI as an early risk marker, thereby supporting clinical follow-up and preventive strategies.

Accordingly, the aim of this study was to evaluate the associations between the AASI derived from 24-h ambulatory blood pressure monitoring (ABPM) and hemodynamic, cardiometabolic, and renal biomarkers, and to identify independent factors associated with the AASI using multivariable regression models. This study is among the few investigations to simultaneously assess the AASI within multivariable models incorporating hemodynamic, cardiometabolic, and renal biomarkers.

## 2. Materials and Methods

This retrospective cross-sectional study was conducted using data from a total of 290 individuals aged 18–65 years who underwent ABPM and were followed at the General Internal Medicine Outpatient Clinic of Gaziantep University Faculty of Medicine between 2020 and 2024. The study was conducted in accordance with the ethical principles of the Declaration of Helsinki. All data were anonymized and analyzed following approval from the GISTU University Non-Interventional Research Ethics Committee (protocol no.: 2025/671, decision no.: 671.48.45, approval date: 18 June 2025).

The primary outcome of this study was to evaluate the association between the AASI and clinical and laboratory parameters. The secondary outcome was to identify independent factors associated with the AASI using multivariable regression analyses.

The study population consisted of 574 individuals aged 18–65 years who underwent ABPM at the General Internal Medicine Outpatient Clinic during the specified study period. None of the individuals had a prior diagnosis of hypertension or were receiving any antihypertensive medications at the time of ABPM evaluation. Based on ABPM findings, participants were subsequently classified as having newly identified hypertension or normotensive blood pressure profiles. To reduce selection bias, only individuals with complete laboratory data were included, comprising hemogram and a comprehensive biochemical panel (fasting glucose, HbA1c, creatinine, estimated glomerular filtration rate [eGFR], sodium, potassium, AST, ALT, HDL cholesterol, LDL cholesterol, triglycerides [TG], total cholesterol, and C-reactive protein [CRP]), as well as complete urinalysis and spot urine albumin-to-creatinine ratio (uACR) results. In accordance with international recommendations, patients with an ABPM recording validity rate of less than 80% were excluded from the study [7]. In addition, pregnant individuals; those with atrial fibrillation or other cardiac arrhythmias; significant valvular heart disease; active infection or overt inflammatory conditions (C-reactive protein (CRP) > 10 mg/L); malignancy; diabetes mellitus; chronic kidney disease; systemic rheumatologic disorders; and individuals receiving regular corticosteroid or immunosuppressive therapy were excluded from the study. Based on these inclusion and exclusion criteria, the final study sample consisted of 290 patients with complete records in the available dataset.

The study population was divided into two groups. The hypertension group comprised individuals with newly identified hypertension based on ABPM measurements. The normotensive group consisted of individuals who underwent ABPM and exhibited normotensive values, defined as systolic blood pressure <135 mmHg and diastolic blood pressure <85 mmHg. The study flow diagram is presented in Figure S1.

To minimize information bias, all laboratory analyses and ABPM measurements were performed using standardized methods routinely applied in clinical practice. Inflammatory hematological indices, including the neutrophil-to-lymphocyte ratio (NLR), platelet-to-lymphocyte ratio (PLR), and monocyte-to-lymphocyte ratio (MLR), were calculated from hemogram data. Estimated glomerular filtration rate (eGFR) was calculated using the 2021 Chronic Kidney Disease Epidemiology Collaboration (CKD-EPI) creatinine equation [8]. Albuminuria was assessed using the uACR [9]. In addition, several cardiometabolic risk indices were calculated from the study database, including the triglyceride–glucose (TyG) index (ln [triglycerides (mg/dL) × fasting glucose (mg/dL)/2]) [10], the TG/HDL cholesterol ratio, non–HDL cholesterol (total cholesterol − HDL cholesterol), the atherogenic index of plasma (AIP: log10 [TG/HDL]) [11], and remnant cholesterol (total cholesterol − LDL cholesterol − HDL cholesterol).

### 2.1. ABPM Measurement Protocol

ABPM was performed in all participants using a standard 24-h protocol. Measurements were obtained with an oscillometric ABPM device that is routinely used in the hospital and has been validated according to international standards. Blood pressure readings were recorded at 30-min intervals during daytime hours and at 60-min intervals during nighttime hours. Prior to device application, all participants were informed about the measurement procedure and were instructed to keep the cuffed arm at heart level and remain still during measurements. ABPM data were analyzed based on complete 24-h recordings. The primary hemodynamic variables derived from ABPM included 24-h mean systolic blood pressure (SBPmean), 24-h mean diastolic blood pressure (DBPmean), maximum systolic blood pressure (SYSmax), minimum systolic blood pressure (SYSmin), maximum diastolic blood pressure (DIAmax), minimum diastolic blood pressure (DIAmin), pulse pressure (PP), mean arterial pressure (MAP), mean heart rate (HRmean), and the AASI [4].

AASI: The AASI was automatically calculated from 24-h SBP and DBP recordings using Formula 1 minus the regression slope of DBP on SBP [4].

In this study, the commonly used 24-h AASI value reported in the literature was employed. All of these parameters were included as dependent and independent variables in the statistical analyses.

### 2.2. Statistical Analysis

Statistical analyses were performed using SPSS (Statistical Package for the Social Sciences) version 22.0 (IBM Corp., Armonk, NY, USA). In descriptive analyses, categorical variables were reported as numbers (*n*) and percentages (%), while continuous variables were expressed as mean ± standard deviation (SD) or median with interquartile range (IQR), along with minimum and maximum values.

The distribution of continuous variables was assessed using the Shapiro–Wilk test. Normality assessment was further supported by visual inspection of histograms and Q–Q plots. Comparisons between two independent groups were performed using the independent samples *t*-test for normally distributed variables and the Mann–Whitney U test for non-normally distributed variables.

Associations between the AASI and continuous variables were evaluated using Pearson or Spearman correlation analyses, as appropriate based on data distribution.

For all tests, the level of statistical significance was set at *p* < 0.05.

Multiple linear regression analysis was performed to identify the independent factors associated with the AASI. Variables were included in the model based on clinical relevance; automated, data-driven selection methods were not utilized. In this context, a hierarchical modeling approach with an additional sensitivity model was applied as follows:

- Model 1 (Hemodynamic Model): The baseline model, including age, maximum systolic blood pressure, and maximum diastolic blood pressure.
- Model 1b (Hemodynamic Sensitivity Model): A sensitivity model including age, mean systolic blood pressure, and mean diastolic blood pressure.
- Model 2 (Hemodynamic + Cardiometabolic Model): Cardiometabolic parameters (LDL cholesterol, TyG index) were added to the baseline model.
- Model 3 (Hemodynamic + Cardiometabolic + Renal Model): uACR as an indicator of albuminuria was incorporated into the model.

SYSmax and DIAmax were selected as the primary hemodynamic variables in the main model because the AASI is mathematically derived from the regression slope between systolic and diastolic blood pressure values and is strongly influenced by the overall blood pressure range across the 24-h period. Maximum systolic and diastolic values reflect peak hemodynamic load and vascular wall stress more directly than averaged measures.

Pulse pressure was not included in the primary model to avoid collinearity, as it is directly derived from systolic and diastolic blood pressure values. Nocturnal dipping parameters, while clinically relevant, primarily reflect circadian blood pressure patterns rather than absolute hemodynamic load and were therefore not selected for the main regression model. To address this, a sensitivity analysis using mean systolic and diastolic blood pressure values (Model 1b) was performed to confirm the robustness of the hemodynamic associations.

All regression models were constructed using the enter method to allow simultaneous evaluation of clinically meaningful variables. Model explanatory power was assessed using the coefficient of determination (R^2^) and adjusted R^2^ values. Assumptions of linear regression were verified, including normal distribution of residuals (histogram and normal P-P plot), homoscedasticity of error variance (scatter plot of standardized residuals versus predicted values), absence of autocorrelation (Durbin-Watson statistic), and lack of multicollinearity, with all variance inflation factor (VIF) values below 3.

Due to the markedly skewed distribution of uACR values, ln-transformed uACR [ln(uACR)] was used in regression analyses. Sensitivity analyses performed using multiple imputation methods demonstrated consistent results, supporting the robustness of the findings.

## 3. Results

A total of 290 individuals were included in the study. Of the participants, 65.2% were female (*n* = 189) and 34.8% were male (*n* = 101). According to ABPM data, hypertension was present in 40.3% of the participants (*n* = 117), while 59.7% were classified as normotensive (*n* = 173).

The demographic characteristics and ambulatory blood pressure measurements of the study population are presented in Table 1. Compared with the normotensive group, individuals in the hypertension group had significantly higher values of age, the AASI, SBPmean, DBPmean, SYSmax, SYSmin, DIAmax, and DIAmin (all *p* < 0.05). PP and MAP were also significantly elevated in hypertensive individuals (both *p* < 0.001). In contrast, no statistically significant difference was observed between the two groups with respect to HRmean (*p* > 0.05).

Table 2 presents the hematological, biochemical, cardiometabolic, and renal parameters of the included participants. Among cardiometabolic indicators, TG levels, the TyG index, the TG/HDL ratio, and AIP were significantly higher in the hypertension group (all *p* < 0.05). In contrast, HDL cholesterol levels were significantly higher in the normotensive group (*p* < 0.001).

Regarding renal function markers, eGFR was significantly lower in the hypertension group (*p* < 0.001), whereas creatinine and uACR levels were markedly higher among hypertensive individuals (*p* < 0.05). No statistically significant differences were observed between the two groups in terms of glucose, HbA1c, sodium, potassium, AST, ALT, LDL cholesterol, total cholesterol, CRP, NLR, PLR, MLR, MPV, non–HDL cholesterol, or remnant cholesterol levels (all *p* > 0.05).

The associations between the AASI and hemodynamic, cardiometabolic, and renal biomarkers are presented in Table 3. The AASI was positively correlated with age (r = 0.197, *p* < 0.001). Among hemodynamic parameters, SYSmax (r = 0.243, *p* < 0.001), SYSmin (r = 0.161, *p* < 0.05), and PP (r = 0.319, *p* < 0.05) showed positive correlations with the AASI, whereas DBPmean (r = −0.117, *p* < 0.05) and DIAmax (r = −0.304, *p* < 0.001) were negatively correlated with the AASI. No significant associations were observed between the AASI and other hemodynamic variables (*p* > 0.05).

Regarding cardiometabolic parameters, glucose (r = 0.134), HbA1c (r = 0.208), and LDL cholesterol (r = 0.126) were positively associated with the AASI, while ALT levels showed a negative correlation (r = −0.180) (all *p* < 0.05). Among cardiometabolic risk indices, the TyG index (r = 0.159, *p* < 0.05), TG/HDL ratio (r = 0.247, *p* < 0.05), non–HDL cholesterol (r = 0.191, *p* < 0.05), AIP (r = 0.285, *p* < 0.05), and remnant cholesterol (r = 0.200, *p* < 0.05) were significantly positively correlated with the AASI. In contrast, HDL cholesterol levels were negatively correlated with the AASI (r = −0.220, *p* < 0.05). TG, total cholesterol, CRP, AST, sodium, potassium, and hematological indices including NLR, PLR, MLR, and MPV showed no significant associations with the AASI (all *p* > 0.05).

With respect to renal biomarkers, the AASI was negatively associated with eGFR (r = −0.217, *p* < 0.001), while uACR levels were positively correlated with the AASI (r = 0.226, *p* < 0.001). No significant association was observed between creatinine levels and the AASI (*p* > 0.05).

In addition to correlation analyses, the associations between the AASI and clinical and cardiometabolic parameters in the 290 included individuals were further evaluated using multivariable linear regression models (Table 4).

In Model 1, which included age and maximum ambulatory blood pressure parameters, the regression model was statistically significant (adjusted R^2^ = 0.357, *p* < 0.001). Age was independently and positively associated with the AASI (β = 0.128, *p* < 0.05). Among hemodynamic variables, SYSmax showed a strong positive association with the AASI (β = 0.586, *p* < 0.001), whereas DIAmax was inversely associated with the AASI (β = −0.645, *p* < 0.001). No evidence of multicollinearity was observed (all VIF values < 2).

To assess the robustness of the hemodynamic associations, a sensitivity analysis was performed using mean ambulatory blood pressure values instead of maximum values (Model 1b). This model was also statistically significant, although with a lower explanatory power (adjusted R^2^ = 0.125, *p* < 0.001). Age remained independently associated with the AASI (β = 0.124, *p* < 0.05). SBPmean was positively associated with the AASI (β = 0.473, *p* < 0.001), while DBPmean demonstrated a significant inverse association (β = −0.495, *p* < 0.001). Collinearity diagnostics indicated acceptable VIF values, confirming the stability of the model.

In Model 2, which incorporated cardiometabolic parameters in addition to age and maximum ambulatory blood pressure values, the regression model remained statistically significant (adjusted R^2^ = 0.346, *p* < 0.001). Age showed a borderline association with the AASI (β = 0.100, *p* = 0.057). Among hemodynamic parameters, SYSmax was positively associated with the AASI (β = 0.560, *p* < 0.001), whereas DIAmax demonstrated a significant inverse association (β = −0.639, *p* < 0.001). In contrast, cardiometabolic markers, including the TyG index (β = 0.082, *p* = 0.144) and LDL cholesterol (β = 0.035, *p* = 0.524), were not independently associated with the AASI. No multicollinearity was detected, as indicated by acceptable VIF values.

In Model 3, renal involvement was assessed by incorporating logarithmically transformed uACR into the cardiometabolic model. This model was statistically significant (adjusted R^2^ = 0.359, *p* < 0.001) and demonstrated a modest increase in explanatory power compared with Model 2. Age was independently associated with the AASI (β = 0.113, *p* = 0.031). SYSmax retained a strong positive association with the AASI (β = 0.501, *p* < 0.001), while DIAmax remained inversely associated (β = −0.614, *p* < 0.001). Notably, ln(uACR) was independently associated with the AASI (β = 0.160, *p* = 0.003). In contrast, TyG index (β = 0.046, *p* = 0.417) and LDL cholesterol (β = 0.050, *p* = 0.364) did not show significant associations. Collinearity diagnostics confirmed the stability of the model.

## 4. Discussion

In this study, we evaluated the factors associated with the AASI using hierarchical multivariable regression models and demonstrated that the AASI is primarily associated with hemodynamic load—particularly SYSmax and DIAmax—and renal microvascular damage as reflected by uACR. Although cardiometabolic markers (including the TyG index and lipid profile) appeared to be associated with the AASI in univariable analyses, these associations lost independence in multivariable models, indicating that the AASI is predominantly associated with hemodynamic and renal axes. Furthermore, the significantly higher AASI observed in hypertensive individuals is consistent with evidence from meta-analyses linking arterial stiffness to target organ involvement [12]. Among non-hemodynamic factors, albuminuria demonstrated the strongest association with the AASI after multivariable adjustment.

In our study, when hematological, biochemical, cardiometabolic, and renal parameters were compared between hypertensive and normotensive individuals, some biomarker changes expected in the context of hypertension were clearly evident, whereas others did not differ significantly between groups. Consistent with the existing literature, hypertensive individuals in our study exhibited significantly higher AIP, TG levels, the TyG index, and the TG/HDL ratio, supporting the well-established association of hypertension with insulin resistance, dyslipidemia, and atherogenic lipoprotein patterns. The higher HDL cholesterol levels observed in the normotensive group further complement this dyslipidemic profile [13,14,15]. In our study, among renal function markers, eGFR was significantly lower in the hypertension group, whereas creatinine and uACR levels were markedly higher in hypertensive individuals. The concomitant reduction in eGFR and elevation of creatinine and uACR in the hypertensive group suggest early adverse effects of hypertension on renal microcirculation. Consistent with previous reports, sustained elevations in blood pressure are known to increase intraglomerular pressure, leading to albuminuria and progressive declines in eGFR, supporting the interpretation that these findings reflect early hypertensive renal involvement [16]. A large-scale cohort study has also demonstrated that albuminuria is associated with an increased risk of cardiovascular events and all-cause mortality, even in individuals with preserved eGFR [17]. Our findings are consistent with contemporary literature suggesting that elevated uACR in hypertensive individuals may reflect not only renal damage but also broader systemic vascular and endothelial dysfunction associated with arterial stiffness [18,19].

When the correlations between the AASI and various demographic, hemodynamic, cardiometabolic, and renal parameters were examined, our findings suggest that hemodynamic stress and age-related biomechanical alterations play a predominant role in the etiology of the AASI, while atherogenic dyslipidemia and early renal injury also make meaningful contributions. Aging is associated with pronounced structural changes in the arterial wall, including elastin fragmentation and increased collagen deposition, which collectively lead to increased arterial stiffness. In this context, the age-related increase in AASI may reflect the cumulative vascular burden and heightened cardiovascular risk observed in older individuals [20]. Consistent with the literature, the AASI in our study was positively associated with age. With respect to hemodynamic markers, our findings indicate that the AASI is particularly sensitive to systolic load. The positive correlation between the AASI and SYSmax likely reflects a physiological consequence of arterial stiffness, as stiffer arteries exhibit a reduced capacity to buffer the maximal distending forces exerted on the arterial wall during systole [12]. Moreover, the strong negative association observed between DIAmax and the AASI in our study reflects reduced compliance of large arteries and impaired Windkessel function. Attenuation of diastolic recoil diminishes the buffering capacity of the arterial system, thereby increasing systolic load and contributing to higher AASI values [21,22]. Our observation of positive associations between glucose and HbA1c levels and the AASI suggests, in line with the existing literature, that glycemic burden may contribute to increased arterial stiffness [23,24]. In contrast, the absence of significant associations between the AASI and conventional cardiometabolic markers such as TG, total cholesterol, and CRP suggests that the AASI is predominantly shaped by hemodynamic load and arterial wall biomechanics. Nevertheless, the significant positive correlations observed between the AASI and atherogenic lipid indices—including LDL cholesterol, the TyG index, the TG/HDL ratio, non-HDL cholesterol, AIP, and remnant cholesterol—support a contributory role of atherogenic dyslipidemia in arterial stiffening [14,15,25,26]. The observed inverse association between eGFR and the AASI aligns with existing evidence suggesting that reductions in renal function parallel increases in arterial stiffness, potentially reflecting shared microvascular and hemodynamic pathways [27]. The significant positive correlation between uACR and the AASI is consistent with robust evidence indicating that albuminuria reflects widespread microvascular injury and systemic endothelial dysfunction [28]. Given that serum creatinine is a relatively late marker of renal injury compared with albuminuria and eGFR, the absence of a significant association between creatinine levels and the AASI is an expected finding.

In this study, the factors associated with the AASI were comprehensively evaluated using a three-step regression modeling approach, and the findings indicate that arterial stiffness is primarily associated with the axes of hemodynamic load, aging, and renal microvascular damage. The persistence of age, SYSmax, and DIAmax as independent factors associated with the AASI in the first two models suggests that arterial stiffness is closely linked to systolic pressure load and loss of diastolic arterial compliance. These results are consistent with the hemodynamic dependency of the AASI reported in the original studies describing this index, as well as in subsequent investigations [3,12,29]. The inverse association between maximum diastolic blood pressure (DIAmax) and the AASI was consistently observed across all three models and is physiopathologically meaningful. This finding is plausible given that the fundamental calculation of the AASI is based on the systolic–diastolic regression slope; increases in peak diastolic values may reduce the slope, thereby resulting in lower AASI values. This observation further supports the concept that the AASI is more sensitive to systolic loading conditions. In addition, the persistence of age as a significant determinant in selected models is consistent with contemporary literature demonstrating that arterial stiffness increases with aging as a consequence of accelerated elastin fiber loss and vascular wall remodeling [20].

In Model 2, the inclusion of cardiometabolic parameters did not meaningfully increase the overall explanatory power of the model. Our findings indicate that cardiometabolic markers contribute more modestly to the AASI compared with hemodynamic variables. This may reflect the dominant influence of hemodynamic load on arterial stiffness in the early stages of hypertensive vascular remodeling. Although some studies have reported that components of the metabolic syndrome may promote arterial stiffness over the long term, the present results indicate that, in this study, the influence of cardiometabolic factors on the AASI is outweighed by the dominant effects of hemodynamic load [30]. Nevertheless, in line with our findings, there are also studies demonstrating that cardiometabolic indicators explain only a limited proportion of the variance in arterial stiffness, with hemodynamic load emerging as the more dominant contributor [31]. Considering that the AASI is an index particularly sensitive to acute pressure loading and to systolic–diastolic interaction dynamics, the absence of cardiometabolic parameters among the variables independently associated with the AASI can be regarded as an expected finding.

In Model 3, uACR was independently associated with the AASI, consistent with existing evidence suggesting that albuminuria may reflect underlying systemic endothelial dysfunction and microvascular damage. [28]. The association between albuminuria and increased arterial stiffness has been previously demonstrated both in hypertensive individuals and in the general population, and even high-normal levels of albuminuria have been reported to adversely affect arterial wall function [18,32]. The notable finding in this model is that, despite cardiometabolic indicators not emerging as independent associated factors, albuminuria assessed by uACR remained significantly and independently associated with the AASI. Clinically, this finding is consistent with a close association between increased arterial stiffness and markers of subclinical renal damage, including micro- and macroalbuminuria. From a clinical perspective, the magnitude of these associations appears modest but meaningful. Based on the fully adjusted model, an increase of 10 mmHg in maximum systolic blood pressure corresponds to an approximate increase of 0.04 units in the AASI. Similarly, a one-unit increase in ln(uACR) is associated with an approximate increase of 0.01 units in the AASI. Although these absolute changes may appear small, the AASI is a dimensionless index with a relatively narrow distribution, and even modest shifts may reflect clinically relevant alterations in arterial stiffness and hemodynamic load. Taken together, these findings support a bidirectional association between albuminuria and arterial stiffness, likely mediated by shared pathophysiological mechanisms such as systemic endothelial dysfunction and microvascular damage.

Overall, our findings indicate that the AASI is strongly associated with hemodynamic loading, age-related alterations of the arterial wall, and subclinical renal injury, whereas classical cardiometabolic parameters do not exert an independent effect on the AASI. These results support the clinical value of the AASI in the early identification of arterial stiffness and underscore the importance of albuminuria-associated subclinical vascular damage in hypertensive individuals. Notably, previous studies have predominantly demonstrated the relationship between arterial stiffness and albuminuria using pulse wave velocity (PWV), which is considered the gold-standard method for the direct assessment of large-artery stiffness [33]. While PWV offers a direct and well-validated measure, its routine clinical use is limited by the requirement for dedicated equipment, operator expertise, and standardized measurement conditions. In contrast, the AASI represents an indirect index of arterial stiffness derived from the systolic-diastolic blood pressure relationship and is influenced by blood pressure variability, which constitutes an important limitation of this measure. In this context, the AASI has also been shown to depend partly on nocturnal dipping status and the ambulatory blood pressure range, methodological characteristics that may influence the calculated index independently of intrinsic arterial stiffness and should therefore be considered when interpreting the AASI as an indirect surrogate marker of arterial stiffness [12]. However, its derivation from routine 24-h ABPM data without additional procedures or cost allows for broad clinical applicability. Accordingly, our findings suggest that the AASI may serve as a practical complementary tool for assessing subclinical cardiorenal interactions, rather than a replacement for PWV, particularly in settings where direct stiffness measurements are not readily available.

## 5. Limitations

This study has several limitations. Its retrospective and cross-sectional design does not allow causal inferences to be drawn. The sample size was relatively limited and based on data from a single center. uACR was assessed based on a single measurement, which may not fully account for intra-individual biological variability. Therefore, the observed association between uACR and the AASI should be interpreted with caution, and repeated measurements in future studies may provide more precise estimates of albuminuria-related vascular risk. In addition, the AASI is mathematically derived from the regression relationship between systolic and diastolic blood pressure and is influenced by blood pressure variability, nocturnal dipping status, and the range of ambulatory blood pressure values. Therefore, its validity as a direct marker of arterial stiffness remains a subject of ongoing debate, and the AASI should be interpreted as an indirect surrogate rather than a definitive measure of large-artery stiffness. Furthermore, direct comparisons with established stiffness indices such as PWV were not available in the present study, which represents an additional methodological limitation. Future prospective studies including simultaneous PWV measurements are warranted to further clarify the clinical role of the AASI.

## 6. Conclusions

In this study, the AASI was shown to be primarily associated with hemodynamic load, and albuminuria was found to be significantly related to the AASI. These findings suggest that the AASI may serve as a practical complementary marker in cardio-renal risk assessment. From a clinical perspective, particular attention should be paid to systolic peak blood pressure when interpreting AASI values. The independent association of albuminuria with the AASI suggests that additional evaluation for subclinical renal damage may be beneficial in individuals with an elevated AASI, even if they are normotensive or have well-controlled hypertension. Therefore, incorporating the AASI together with renal markers such as albuminuria may be appropriate for a comprehensive assessment of cardiorenal risk.

Given that the AASI can be easily derived from ABPM, is non-invasive, and does not require additional cost, its potential use as a practical tool for the early detection of renal and vascular damage is strengthened. Future prospective and long-term studies should evaluate the longitudinal relationships between changes in the AASI and cardiometabolic and renal parameters, and further clarify the predictive value of integrated hemodynamic–cardiometabolic–renal models for cardiovascular and renal outcomes.

## Figures and Tables

**Table 1:** Demographic characteristics and ambulatory blood pressure parameters of the study population.

Characteristics (*n* = 290)	Total (*n* = 290) Mean ± SD (Median, IQR; Min–Max)	Hypertension (*n* = 117) Mean ± SD (Median, IQR; Min–Max)	Normotensive (*n* = 173) Mean ± SD (Median, IQR; Min–Max)	*p*
Age (years)	46.5 ± 12.3 (48, 18; 18–65)	48.45 ± 11.6 (49, 18; 18–65)	45.3 ± 12.60 (45, 18; 18–65)	<0.05 *
AASI	0.467 ± 0.156 (0.46, 0.192; 0.00–0.87)	0.514 ± 0.176 (0.510, 0.270; 0.04–0.87)	0.438 ± 0.133 (0.440, 0.180; 0.00–0.80)	<0.001 *
SBPmean (mmHg)	130.24 ± 15.39 (130.2, 22; 98–186)	144.45 ± 12.31 (143, 14; 110–186)	120.63 ± 8.09 (122, 14; 98–134)	<0.001 *
DBPmean (mmHg)	76.51 ± 10.18 (76, 12.25; 55–114)	83.56 ± 10.50 (84, 13; 58–114)	71.74 ± 6.66 (72, 10; 55–84)	<0.001 *
SYSmax (mmHg)	168.42 ± 22.2 (167.4, 28; 95–237)	184.49 ± 20.34 (182, 28; 95–236)	155.87 ± 14.73 (157, 23; 118–187)	<0.001 *
SYSmin (mmHg)	96.04 ± 14.12 (92.5, 17; 72–152)	105.08 ± 16.09 (103, 26; 72–152)	89.94 ± 8.20 (89, 12; 72–113)	<0.001 *
DIAmax (mmHg)	105.16 ± 18.28 (103, 26; 68–145)	112.27 ± 15.07 (112, 22; 70–145)	100.36 ± 18.7 (97, 24; 68–113)	<0.001 *
DIAmin (mmHg)	51.40 ± 8.89 (50, 10; 40–102)	55.21 ± 11.25 (53, 14; 40–102)	48.83 ± 5.56 (48, 9; 40–68)	<0.001 *
PP (mmHg)	53.7 ± 9.6 (51, 12; 38–85)	60.6 ± 10.04 (60, 16; 43–85)	49 ± 5.76 (49, 7; 38–72)	<0.001 *
MAP (mmHg)	94.42 ± 11.27 (93.5, 15.2; 71–133)	103.86 ± 10.07 (105, 11; 76–140)	88.03 ± 6.59 (90, 9.5; 71–99)	<0.001 *
HRmean (beats/min)	78.46 ± 8.78 (78, 11; 42–106)	79.14 ± 10.23 (79, 14; 42–105)	78.01 ± 7.83 (78, 10; 60–106)	>0.05

*p* value: Independent samples *t*-test. The asterisk (*) indicates statistically significant values (*p* < 0.05). AASI: Ambulatory Arterial Stiffness Index, SBPmean: Mean Systolic Blood Pressure, DBPmean: Mean Diastolic Blood Pressure, SYSmax: Maximum Systolic Blood Pressure, SYSmin: Minimum Systolic Blood Pressure, DIAmax: Maximum Diastolic Blood Pressure, DIAmin: Minimum Diastolic Blood Pressure, PP: Pulse Pressure, MAP: Mean Arterial Pressure. HRmean: Heart Rate Mean.

**Table 2:** Baseline laboratory findings, cardiometabolic and renal indicators.

Characteristics (*n* = 290)	Total (*n* = 290) Mean ± SD (Median, IQR; Min–Max)	Hypertension (*n* = 117) Mean ± SD (Median, IQR; Min–Max)	Normotensive (*n* = 173) Mean ± SD (Median, IQR; Min–Max)	*p*
Glucose (mg/dL)	95.7 ± 14.3 (94, 20; 56–124)	97.95 ± 14.4 (96, 23; 65–124)	94.2 ± 14.1 (92, 18; 56–124)	>0.05
HbA1c (%)	5.44 ± 0.51 (5.4, 0.7; 3.9–6.4)	5.49 ± 0.53 (5.50, 0.70; 4.20–6.4)	5.41 ± 0.49 (5.40, 0.7; 3.90–6.4)	>0.05
Creatinine (mg/dL)	0.781 ± 0.249 (0.74, 0.27; 0.40–2.70)	0.843 ± 0.307 (0.80, 0.355; 0.40–2.70)	0.740 ± 0.193 (0.72, 0.210; 0.43–2.00)	<0.05 *
eGFR (mL/min/1.73 m^2^)	100.06 ± 18.56 (102, 23; 32–139)	95.15 ± 19.54 (99, 26; 32–130)	103.28 ± 17.19 (104, 23; 49–139)	<0.001 *
Sodium (mmol/L)	138.77 ± 2.56 (139, 2; 128–146)	138.92 ± 2.71 (139, 1; 128–145)	138.67 ± 2.46 (139, 3; 128–146)	>0.05
Potassium (mmol/L)	4.29 ± 0.38 (4.29, 0.22; 3.09–5.38)	4.26 ± 0.39 (4.20, 0.50; 3.20–5.20)	4.32 ± 0.38 (4.30, 0.52; 3.09–5.38)	>0.05
AST (U/L)	23.69 ± 14.56 (21, 5; 10–214)	25.40 ± 20.30 (21, 8; 12–214)	22.54 ± 8.75 (21, 9; 10–82)	>0.05
ALT (U/L)	25.60 ± 17.59 (21, 9; 8–172)	26.90 ± 20.63 (22, 12; 8–172)	24.73 ± 15.25 (21, 16; 8–147)	>0.05
HDL cholesterol (mg/dL)	49.79 ± 13.92 (48.5, 9; 29–95)	44.04 ± 9.00 (42, 14; 30–66)	54.34 ± 15.42 (54, 20; 29–95)	<0.001 *
LDL cholesterol (mg/dL)	135.26 ± 31.90 (135, 24; 63–228)	134.34 ± 32.42 (137, 48; 63–213)	135.89 ± 31.64 (133, 49; 70–228)	>0.05
Triglycerides (TG) (mg/dL)	173.30 ± 114.16 (142.5, 100; 23–804)	193.49 ± 134.56 (158.5, 115; 52–804)	160.18 ± 96.89 (136.5, 110; 23–736)	<0.05 *
Total cholesterol (mg/dL)	220.61 ± 45.22 (219.9, 31.9; 118–339.4)	217.33 ± 41.02 (220, 54; 139–313)	223.11 ± 48.32 (219.5, 70; 118–339)	>0.05
CRP (mg/L)	3.60 ± 2.5 (3, 3.8; 0–9.40)	3.66 ± 2.38 (3, 3.1; 0.30–9.40)	3.71 ± 2.61 (3, 3.50; 0–9.30)	>0.05
NLR	2.06 ± 1.13 (1.81, 0.55; 0.30–10.48)	1.98 ± 0.82 (1.823, 0.93; 0.79–5.95)	2.12 ± 1.30 (1.797, 1.03; 0.30–10.48)	>0.05
PLR	117.19 ± 46.15 (112.2, 20; 12.7–413.2)	115.31 ± 44.13 (112.9, 41.7; 12.7–319.5)	118.44 ± 47.52 (110.7, 48.5; 32.2–413.2)	>0.05
MLR	0.252 ± 0.115 (0.227, 0.07; 0.05–1.12)	0.2429 ± 0.1050 (0.227, 0.115; 0.05–0.82)	0.2577 ± 0.1213 (0.2279, 0.118; 0.11–1.12)	>0.05
MPV (fL)	10.57 ± 0.93 (10.50, 1.50; 8.40–13.90)	10.65 ± 1.02 (10.60, 1.20; 8.40–13.90)	10.51 ± 0.85 (10.50, 1.00; 8.70–13.40)	>0.05
uACR (mg/g)	90.16 ± 229.28 (0, 62.5; 0–2000)	197.4 ± 318.6 (60, 259; 0–2000)	18.26 ± 84.92 (0, 84.92; 0–1000)	<0.001 *
TyG index	8.90 ± 0.64 (8.86, 0.81; 6.99–11.04)	9.04 ± 0.65 (8.905, 0.85; 7.74–11.04)	8.63 ± 0.62 (8.47, 0.83; 6.99–10.70)	<0.05 *
TG/HDL ratio	3.96 ± 3.05 (3.20, 3.12; 0.32–19.97)	4.65 ± 3.30 (3.76, 3.24; 1.29–19.97)	3.43 ± 2.75 (2.619, 2.10; 0.32–18.61)	<0.05 *
Non–HDL cholesterol (mg/dL)	170.75 ± 42.02 (167.2, 58.4; 81.4–281.4)	173.35 ± 40.98 (176.2, 52; 91.20–281.40)	168.77 ± 42.99 (164.4, 55; 81.40–271.40)	>0.05
AIP	0.13 ± 0.29 (0.14, 0.41; −0.85–0.94)	0.23 ± 0.03 (0.21, 0.33; −0.25–0.94)	0.06 ± 0.03 (0.05, 0.45; −0.85–0.91)	<0.05 *
Remnant cholesterol (mg/dL)	35.16 ± 20.39 (29.80, 26.25; 4.6–123.8)	38.00 ± 21.13 (31.80, 24; 12.60–123.80)	33.00 ± 19.70 (27.20, 17.60; 4.60–115.40)	>0.05

*p* values were obtained using the independent samples *t*-test for normally distributed variables and the Mann–Whitney U test for non-normally distributed variables, as appropriate. The asterisk (*) indicates statistically significant values (*p* < 0.05). TG: triglycerides; NLR: neutrophil-to-lymphocyte ratio; PLR: platelet-to-lymphocyte ratio; MLR: monocyte-to-lymphocyte ratio; uACR: spot urine albumin-to-creatinine ratio.

**Table 3:** Correlations between the AASI and hemodynamic, cardiometabolic, and renal biomarkers.

Characteristics (*n* = 290)	r	*p*
Age (years)	0.197	<0.001 *
SBPmean	0.110	>0.05
DBPmean	−0.117	<0.05 *
SYSmax	0.243	<0.001 *
SYSmin	0.161	<0.05 *
DIAmax	−0.304	<0.001 *
DIAmin	0.101	>0.05
PP	0.319	<0.001 *
MAP	−0.020	>0.05
HRmean	−0.071	>0.05
Glucose (mg/dL)	0.134	<0.05 *
HbA1c (%)	0.208	<0.05 *
Creatinine (mg/dL)	−0.011	>0.05
eGFR (mL/min/1.73 m^2^)	−0.217	<0.001 *
Sodium (mmol/L)	−0.031	>0.05
Potassium (mmol/L)	0.067	>0.05
AST (U/L)	−0.068	>0.05
ALT (U/L)	−0.180	<0.05 *
HDL cholesterol (mg/dL)	−0.220	<0.05 *
LDL cholesterol (mg/dL)	0.126	<0.05 *
Triglycerides (TG) (mg/dL)	0.091	>0.05
Total cholesterol (mg/dL)	0.108	>0.05
CRP (mg/L)	−0.013	>0.05
NLR	−0.013	>0.05
PLR	−0.045	>0.05
MLR	−0.047	>0.05
MPV (fL)	0.097	>0.05
uACR (mg/g)	0.226	<0.001 *
TyG index	0.159	<0.05 *
TG/HDL ratio	0.247	<0.05 *
Non–HDL cholesterol (mg/dL)	0.191	<0.05 *
AIP	0.285	<0.05 *
Remnant cholesterol (mg/dL)	0.200	<0.05 *

*p* values were obtained using the Pearson correlation test for normally distributed variables and the Spearman correlation test for non-normally distributed variables, as appropriate. The asterisk (*) indicates statistically significant values (*p* < 0.05). SBPmean: mean systolic blood pressure; DBPmean: mean diastolic blood pressure; SYSmax: maximum systolic blood pressure; SYSmin: minimum systolic blood pressure; DIAmax: maximum diastolic blood pressure; DIAmin: minimum diastolic blood pressure; PP: pulse pressure; MAP: mean arterial pressure; HRmean: mean heart rate; TG: triglycerides; NLR: neutrophil-to-lymphocyte ratio; PLR: platelet-to-lymphocyte ratio; MLR: monocyte-to-lymphocyte ratio; uACR: spot urine albumin-to-creatinine ratio.

**Table 4:** Results of multivariable linear regression analyses for Models 1–3 evaluating the effects of hemodynamic, cardiometabolic, and renal markers on the AASI.

Model	Variable	B	Std. Error	β	t	*p*-Value	95% CI for B	VIF
1 Hemodynamic (Max BP)	Constant	0.281	0.061	—	4.646	<0.001 *	0.162–0.400	NA
	Age	0.002	0.001	0.128	2.677	0.008 *	0.000–0.003	1.036
	SYSmax	0.004	0.000	0.586	10.069	<0.001 *	0.003–0.005	1.524
	DIAmax	−0.005	0.000	−0.645	−11.236	<0.001 *	−0.006–−0.005	1.480
	Adjusted R^2^ 0.357							
1b Hemodynamic (Mean BP)	Constant	0.351	0.077	—	4.563	<0.001 *	0.200–0.502	NA
	Age	0.002	0.001	0.124	2.161	0.032 *	0.000–0.003	1.082
	SBPmean	0.005	0.001	0.473	5.122	<0.001 *	0.003–0.007	2.822
	DIAmean	−0.008	0.001	−0.495	−5.467	<0.001 *	−0.010–−0.005	2.708
	Adjusted R^2^ 0.125							
2 Hemodynamic + Cardiometabolic	Constant	0.100	0.120	—	0.830	0.408	−0.137–0.336	NA
	Age	0.001	0.001	0.100	1.911	0.057	0.000–0.003	1.088
	SYSmax	0.004	0.000	0.560	9.000	<0.001 *	0.003–0.005	1.548
	DIAmax	−0.005	0.001	−0.639	−10.415	<0.001 *	−0.006–−0.004	1.500
	TyG	0.020	0.014	0.082	1.467	0.144	−0.007–0.047	1.236
	LDL	0.000	0.000	0.035	0.639	0.524	0.000–0.001	1.220
	Adjusted R^2^ 0.346							
3 Hemodynamic + Cardiometabolic + Renal	Constant	0.197	0.123	—	1.599	0.111	−0.046–0.439	NA
	Age	0.001	0.001	0.113	2.174	0.031 *	0.000–0.003	1.093
	SYSmax	0.004	0.000	0.501	7.720	<0.001 *	0.003–0.004	1.711
	DIAmax	−0.005	0.001	−0.614	−10.049	<0.001 *	−0.006–−0.004	1.514
	TyG	0.011	0.014	0.046	0.814	0.417	−0.016–0.038	1.298
	LDL	0.000	0.000	0.050	0.909	0.364	0.000–0.001	1.231
	ln(uACR)	0.010	0.004	0.160	2.953	0.003 *	0.003–0.017	1.190
	Adjusted R^2^ 0.359							

The asterisk (*) indicates statistically significant values (*p* < 0.05). Model 1: Hemodynamic (maximum blood pressure) model. Model 1b: Hemodynamic (mean blood pressure) sensitivity model. Model 2: Hemodynamic plus cardiometabolic model. Model 3: Hemodynamic plus cardiometabolic plus renal model. β: standardized regression coefficient; Std. Error: standard error; CI: confidence interval; R^2^: coefficient of determination.

## Data Availability

The datasets generated and/or analyzed during the current study are not publicly available due to institutional data protection policies but are available from the corresponding author on reasonable request.

## Supplementary Materials

The following supporting information can be downloaded at https://www.mdpi.com/article/10.3390/jcm15041498/s1, Figure S1: Flowchart illustrating the selection process of the study population and group allocation based on ambulatory blood pressure monitoring (ABPM) results.
