# Supplementary material for: Albuminuria as a Key Factor Associated with Ambulatory Arterial Stiffness: A Hierarchical Multivariable Analysis
Source: J Clin Med. 2026 Feb 14;15(4):1498. doi: 10.3390/jcm15041498 (PMC12941870; doi:10.3390/jcm15041498)
Supplement: Supplementary file 1 [file jcm-15-01498-s001.zip › jcm-4130981-supplementary.pdf]

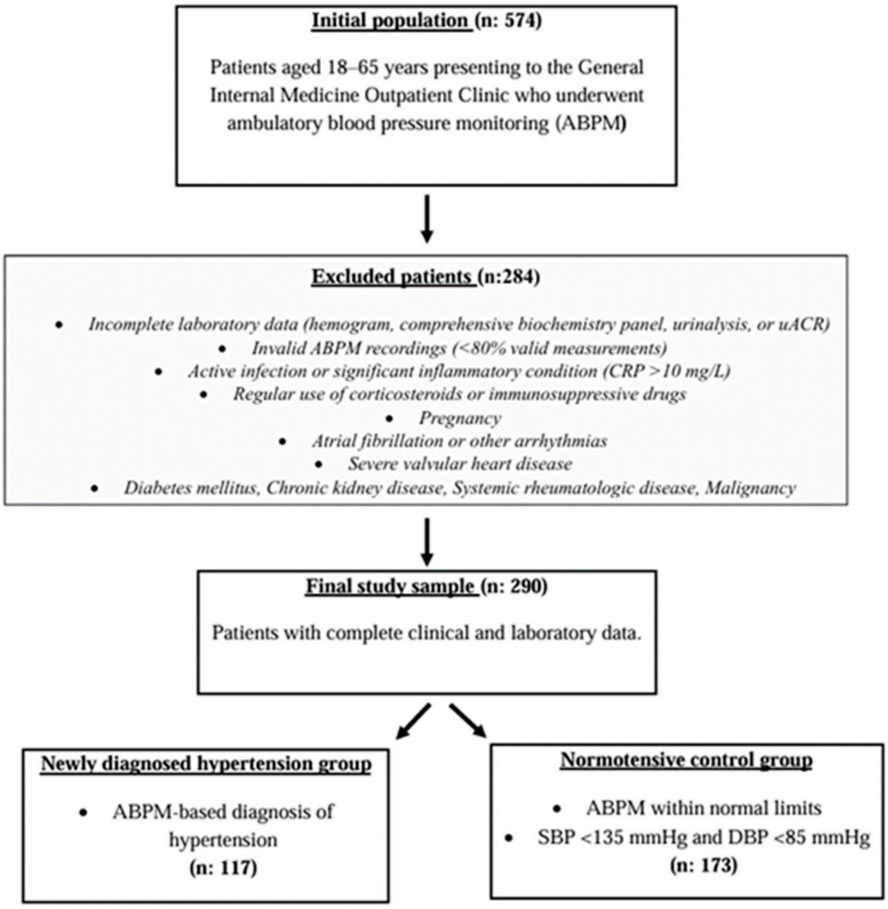

Figure S1. Flowchart illustrating the selection process of the study population and group allocation based on ambulatory blood pressure monitoring (ABPM) results.
